# PtosisDiffusion: a training-free workflow for precisely predicting post-operative appearance in blepharoptosis patients based on diffusion models

**DOI:** 10.3389/fcell.2024.1459336

**Published:** 2024-10-30

**Authors:** Shenyu Huang, Jiajun Xie, Boyuan Yang, Qi Gao, Juan Ye

**Affiliations:** ^1^ Eye Center, The Second Affiliated Hospital, School of Medicine, Zhejiang University, Zhejiang Provincial Key Laboratory of Ophthalmology, Zhejiang Provincial Clinical Research Center for Eye Diseases, Zhejiang Provincial Engineering Institute on Eye Diseases, Hangzhou, Zhejiang, China; ^2^ Department of Mechanical Science and Engineering, University of Illinois at Urbana-Champaign, Urbana, IL, United States

**Keywords:** deep learning, diffusion model, blepharoptosis, post-operative appearance, prediction

## Abstract

**Purpose:**

This study aims to develop a diffusion-based workflow to precisely predict postoperative appearance in blepharoptosis patients.

**Methods:**

We developed PtosisDiffusion, a training-free workflow that combines face mesh with ControlNet for accurate post-operative predictions, and evaluated it using 39 preoperative photos from blepharoptosis patients. The performance of PtosisDiffusion was compared against three other diffusion-based methods: Conditional Diffusion, Repaint, and Dragon Diffusion.

**Results:**

PtosisDiffusion demonstrated superior performance in subjective evaluations, including overall rating, correction, and double eyelid formation. Statistical analyses confirmed that PtosisDiffusion achieved the highest overlap ratio (0.87 ± 0.07) and an MPLPD ratio close to 1 (1.01 ± 0.10). The model also showed robustness in extreme cases, and ablation studies confirmed the necessity of each model component.

**Conclusion:**

PtosisDiffusion generates accurate postoperative appearance predictions for ptosis patients using only preoperative photographs. Among the four models tested, PtosisDiffusion consistently outperformed the others in both subjective and statistical evaluation.

## 1 Introduction

Blepharoptosis (Ptosis) is characterized by an abnormally low upper eyelid margin in the primary gaze, which narrows the eye opening and partially covers the eye (Finsterer, 2003). Unilateral or bilateral ptosis can impair appearance and visual function, significantly affecting quality of patient life by causing amblyopia and increased anxiety and depression related to appearance, thereby impacting overall patient wellbeing. The primary treatment for managing ptosis is surgery (Bacharach et al., 2021). Surgical correction of ptosis is recommended not only for cosmetic improvement but also to prevent visual impairments (Salamah et al., 2022). However, ptosis surgery is highly personalized, with the type of procedure determined by the underlying cause of ptosis, its severity, and the function of the levator muscle. This personalized approach, combined with the potential for unexpected surgical outcomes, can increase patient anxiety and depression, and may even reduce patient confidence in decision-making. Therefore, accurate prediction the of postoperative appearance is essential for the success of ptosis surgery (Koka and Patel, 2019).

Accurate predictions provide surgeons with visual feedback on the anticipated changes, aiding in the optimization of surgical plans and the precise adjustment of eyelid positioning to correct any residual deformities. This approach not only improves the precision and effectiveness of the surgery but also enhances patient understanding and confidence in the outcomes. There have been attempts to predict postoperative results in ptosis surgery. Mawatari et al. utilize Adobe Photoshop for predicting levator resection images (Mawatari and Fukushima, 2016) and employ mirror image processing software for ptosis surgery (Mawatari et al., 2021). However, this method is limited by the subjective nature of manual image manipulation and operator variability. Sun el al employed a Generative Adversarial Network (Goodfellow et al., 2014) (GAN) trained on paired pre- and post-surgery data to perform image translation tasks for ptosis prediction (Sun et al., 2022). The GAN approach have been used in image translation tasks widely (Song et al., 2023; Armanious et al., 2020). Though more automated, it faces several constraints. It requires paired data of pre- and post-operative images, which are difficult to acquire. Additionally, GANs are known for training instability and artifacts in generated images. Some results exhibit unrealistic artifacts in the center of the eyebrow.

Recently, diffusion-based methods (Ho et al., 2020; Nichol and Dhariwal, 2021; Rombach et al., 2021) have demonstrated significant success in generating realistic images. Leveraging these advancements, we developed a workflow named PtosisDiffusion, which can accurately predict postoperative outcomes in blepharoptosis without requiring additional model training. We compared PtosisDiffusion with three other diffusion-based methods using human evaluation and abstract statistical analysis. Our results indicate that PtosisDiffusion outperforms the other three methods.

Our primary contributions are as follows.

• **Unpaired Data Requirement:** In contrast to other image translation methods, our model operates without the need for paired data.

• **Training Efficiency:** Our model functions effectively without necessitating additional training.

• **State-of-the-Art Performance:** Our model achieves state-of-the-art results.

• **New Evaluation Parameters Proposed:** We propose two evaluation parameters that are agnostic to real-world measurements and are solely based on image analysis.

## 2 Methods

We began with the quantification of facial attributes, followed by a brief introduction to diffusion methods and a detailed explanation of each individual method.

### 2.1 Ptosis attributes measurements

**FIGURE 1:**
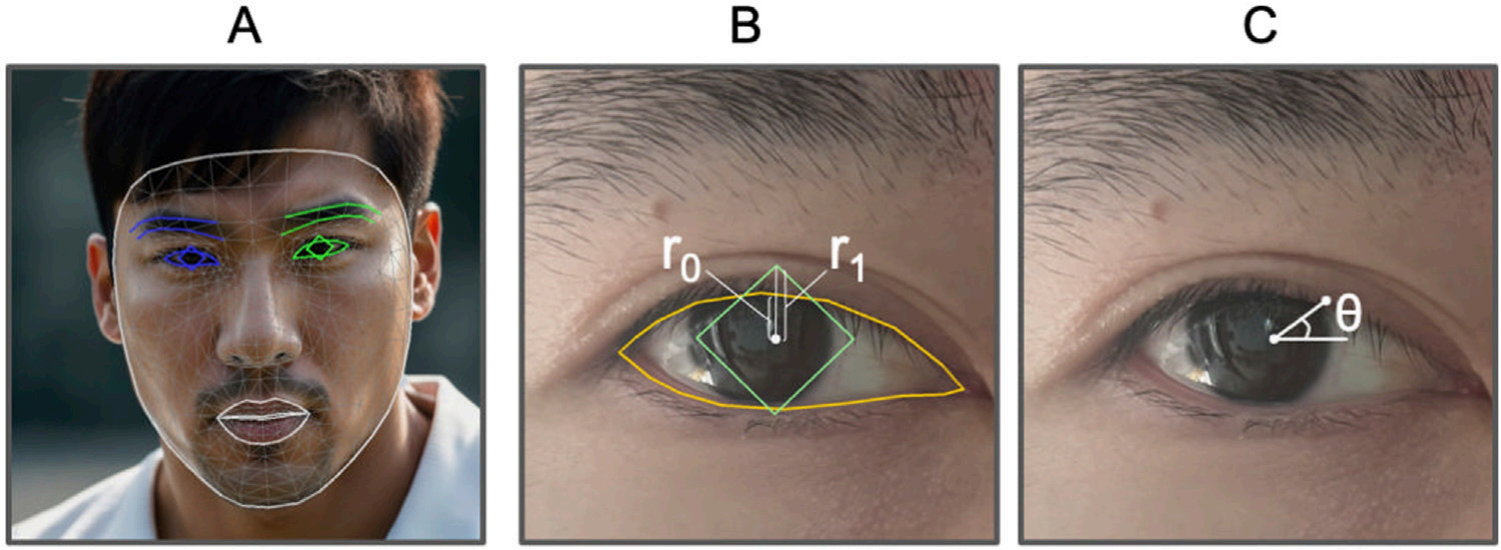
Face attributes illustration. **(A)** Example of face mesh (human face generated by AI). **(B)** $MRR=r_{0}/r_{1}$ where $r_{0}$ indicates pixel distance from iris center point to upper lid point and $r_{1}$ indicates pixel distance from iris center point to iris top point. **(C)** MPLPD indicates pixel distance from iris center point to lid point for angle θ .

To advance the study of ptosis, it is crucial to develop efficient methods for quantifying various clinical measurements. Traditionally, this process requires the use of a reference object with known dimensions, such as a ruler or a sticker of predetermined size, placed adjacent to the face. While this approach provides accurate measurements, the availability of such reference objects is limited to specific circumstances, thereby restricting the broader application of these clinical measurements. Leveraging artificial intelligence (AI) offers a promising avenue for achieving this goal. Specifically, the use of face mesh, a machine learning model that accurately maps facial landmarks, presents a robust starting point. Face mesh and iris detection (Kartynnik et al., 2019; Ablavatski et al., 2020) detects 468 face landmark 3D points and iris locations, see Figure 1A. Comparing with traditional face landmarks detection with typically 68 points, it achieves better accuracy and offers more flexible face landmarks choices.

Inspired by the marginal reflex distance-1 (MRD1) (Bodnar et al., 2016) and mid-pupil lid distances (MPLDs) (Morris et al., 2011), we define analogous quantities for measuring ptosis attributes. First, we introduce the marginal reflex ratio (MRR), see Figure 1B. This ratio is determined by two vertical distances: the first is the distance along the *y*-axis from the center of the pupil to the top edge of the pupil’s bounding box, and the second is the distance from the center of the pupil to the top edge of the upper eyelid. Next, we define mid-pupil lid pixel distances (MPLPDs), see Figure 1C, which are similar to MPLDs but measured in pixels. These two criteria are utilized for guiding the models and for evaluating their performance metrics.

### 2.2 Diffusion methods

Diffusion models are a class of generative models that have gained significant attention for their ability to produce high-quality synthetic samples, including images, audio, and text. In the realm of medical imaging, diffusion methods have been applied to tasks such as classification, segmentation, denoising and reconstruction (Wolleb et al., 2022; Sanchez et al., 2022; Chung and Ye, 2022; Friedrich et al., 2023). Compared to other generative methods, diffusion models excel in generation quality and training stability. Numerous studies have expanded their generative capabilities, including control over the structure of generated results and the manipulation of color schemes (Ruiz et al., 2023; Zhang et al., 2023; Hu et al., 2021).

Recent stable diffusion models trained on billions of real images have achieved remarkable results (Rombach et al., 2021). We leverage this capability for post-operative prediction. We developed a workflow called PtosisDiffusion based on face mesh (Kartynnik et al., 2019; Ablavatski et al., 2020) and ControlNet (Zhang et al., 2023) for accurate post-operative prediction. Additionally, we compared this workflow with three other diffusion-based methods, namely, Conditional Diffusion (Song et al., 2020), Repaint (Lugmayr et al., 2022) and Dragon Diffusion (Mou et al., 2023).

#### 2.2.1 Denoising diffusion probabilistic model (DDPM)

**FIGURE 2:**
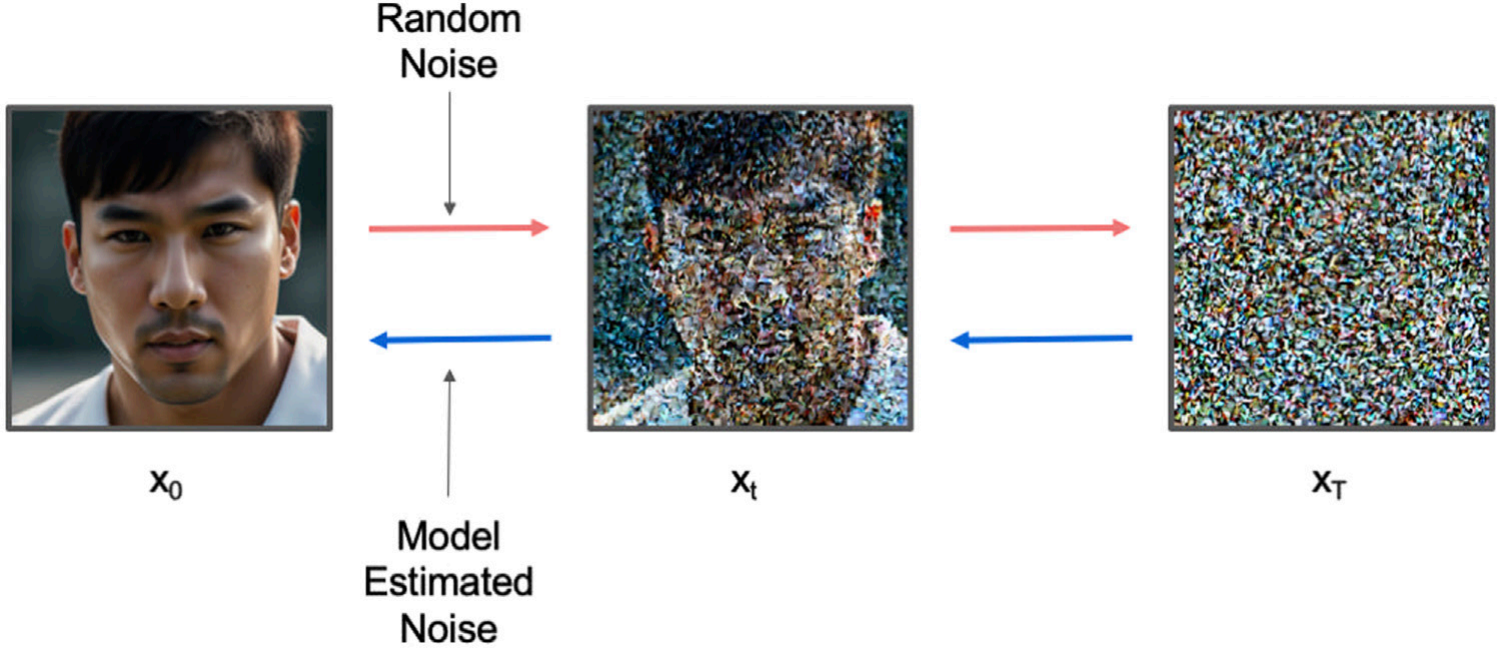
The workflow for DDPM. Red arrow indicates forward process which gradually adds random Gaussian noise to the image. Blue arrow indicates reverse process which gradually denoises the image.

As the foundational diffusion model, Denoising Diffusion Probabilistic Models (Ho et al., 2020) (DDPM) serve as the basis for all subsequent diffusion-based methods. DDPM consists of two processes: the forward process, which repeatedly adds small amounts of Gaussian noise to the sample $x_{0}$ until it becomes random noise $x_{T}$ , and the reverse process, which gradually denoises $x_{T}$ back to the original sample $x_{0}$ based on the model estimated noise, see Figure 2. Assuming the data distribution of $x_{t}$ is $q\left(x_{t}\right)$ . The forward process can be characterized by Equation 1:

$$q\left(x_{t}|x_{t-1}\right)=N\left(x_{t};\sqrt{1-\beta_{t}}x_{t-1},\beta_{t}I\right),$$ (1)

where $\beta_{t}\in \left(0,1\right)$ is a predefined hyper-parameter. Moreover, a sample at any given time t can be characterized as Equation 2:

$$x_{t}=\sqrt{{\bar{\alpha }}_{t}}x_{0}+\sqrt{1-{\bar{\alpha }}_{t}}\epsilon_{t},\epsilon_{t}∼N\left(0,I\right)$$ (2)

where $\alpha_{t}=1-\beta_{t},{\bar{\alpha }}_{t}=\prod_{i=1}^{T}\alpha_{i}=\prod_{i=1}^{T}\left(1-\beta_{i}\right)$ . The reverse process is learnable and characterized by Equation 3:

$$p_{\theta }\left(x_{t-1}|x_{t}\right)=N\left(x_{t-1};\mu_{\theta }\left(x_{t},t\right),\Sigma_{\theta }\left(x_{t},t\right)\right)$$ (3)

and further reduced to Equation 4:

$$x_{t-1}=\frac{1}{\sqrt{\alpha_{t}}}\left(x_{t}-\frac{1-\alpha_{t}}{\sqrt{1-{\bar{\alpha }}_{t}}}\epsilon_{\theta }\left(x_{t},t\right)\right)+\sigma_{t}z,$$ (4)

where z∼N(0,I) , $\sigma_{t}$ is a constant derived from $\beta_{t}$ and $\epsilon_{\theta }$ is an estimation of $\epsilon_{t}$ .

During training, a U-Net model is trained to estimate $\epsilon_{\theta }$ . The loss function can be derived as Equation 5:

$$E_{t∼U\left[\left[1,T\right]\right],x_{0}∼q\left(x_{0}\right),\epsilon ∼N\left(0,I\right)}\left[\lambda \left(t\right){\left\|\epsilon -\epsilon_{\theta }\left(x_{t},t\right)\right\|}^{2}\right]$$ (5)

where λ(t) is a positive weighting function, $x_{t}$ is computed by Equation 2, t∼U[[1,T]] is a uniform distribution over the set {1,2,…,T} .

During inference, we sample $x_{T}$ from Gaussian distribution. Following reverse process, the noise $\epsilon_{\theta }$ at each step t is estimated and subtracted from $x_{t}$ . At final step, we obtain a clean image $x_{0}$ .

#### 2.2.2 Conditional Diffusion

Conditional Diffusion leverages a probabilistic model to generate images guided by predefined conditions, such as postoperative targets. By incorporating prior information, the diffusion process is directed to produce images that match desired outcomes. This technique is often used for tasks like image synthesis and inpainting, ensuring that the generated images meet specific criteria. Conditional diffusion model generates result under condition label y . Using Bayesian rule, it can be shown (Song et al., 2020) that conditional diffusion can generate images using an unconditional diffusion model, augmented by an additional term $\nabla_{x_{t}}\log p_{t}\left(y|x_{t}\right)$ , where $x_{t}$ is the image sample at step t , to account for the conditional information. We use the target MRR as condition label and create a Gaussian distribution for $p_{t}\left(y|x_{t}\right)$ . Then the conditional term becomes $-\nabla_{x_{t}}{\left(y-y_{0}\right)}^{2}$ where y is the measured MRR for image $x_{t}$ and $y_{0}$ is the target MRR. This approach enables the generation of images with an MRR value close to the desired target, ensuring accurate prediction of postoperative outcomes.

#### 2.2.3 Repaint

Repaint (Lugmayr et al., 2022) is a variation of the diffusion inpainting technique that focuses on editing image content within a specified mask region. During the denoising process, it systematically adds noise to the masked areas, ensuring that their latent distribution gradually aligns with that of the adjacent unmasked sections. Repaint iteratively refines targeted regions, blending them seamlessly with the surrounding image, achieving the desired modifications while maintaining coherence with the unedited sections. This approach is frequently used for tasks requiring selective changes, such as image correction or restoration. In this study, it ensures that edited areas of the eyelid are integrated smoothly into the overall postoperative prediction.

#### 2.2.4 Dragon Diffusion

Dragon Diffusion (Mou et al., 2023) enables drag-style manipulation through gradient guidance and cross-attention guidance, everaging advanced mathematical principles such as attention mechanisms and gradient descent. This approach allows for dynamic adjustments in image generation, facilitating tasks such as object movement, resizing, appearance replacement, and object pasting on diffusion models. The ability to manipulate image elements in real-time makes Dragon Diffusion particularly valuable in applications such as augmented reality and image editing, where precise control over visual content is essential. In this study, we utilize a face mesh to detect key points on the upper eyelid, and manually determine the targeted dragging points. This allows for fine-tuned adjustments to the eyelid’s position, which is crucial for generating accurate predictions of postoperative appearance.

#### 2.2.5 PtosisDiffusion

We designed a PtosisDiffusion workflow specifically designed for ptosis post-operative prediction, leveraging ControlNet to provide additional control over the image generation process. ControlNet introduces external conditioning information (such as the edge map) into the diffusion process via zero-convolution, utilizing convolutional neural networks (CNNs) to influence the latent space of the diffusion model, enabling more targeted and constrained generation. A common implementation of ControlNet involves integrating Canny edge detection (Canny, 1986) as a conditioning method. Canny edge detection is a widely-used algorithm that identifies boundaries within an image by detecting areas of rapid intensity change, producing a binary image where edges are highlighted. This representation offers a clear and concise depiction of the structural outlines within the original image. The pre-trained ControlNet model guides the diffusion model to generate images that have similar Canny edge detection results.

**FIGURE 3:**
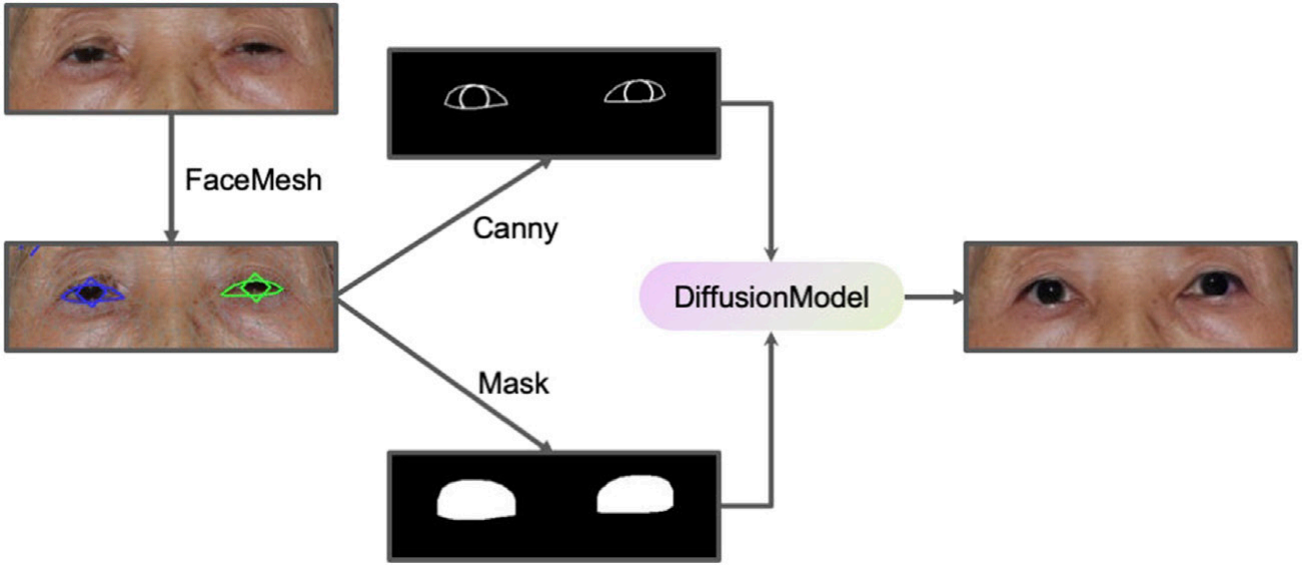
The workflow of PtosisDiffusion.

By employing face mesh technology, we developed a robust workflow for accurate ptosis postoperative prediction. Initially, the face mesh is used to detect the patient’s eye contour and iris location, from which a Canny edge image and a binary mask are generated. The Canny edge image reflects the appropriate MRR value and serves as input for ControlNet diffusion inpainting, while the binary mask is utilized in the inpainting process. This methodology ensures that the generated images retain the desired structural characteristics, which are crucial for accurate and reliable predictions of postoperative outcomes. Furthermore, this approach has applications in other fields requiring precise image manipulation, such as cosmetic surgery and facial reconstruction. A schematic representation of this workflow is illustrated in Figure 3.

## 3 Experiment results

**FIGURE 4:**
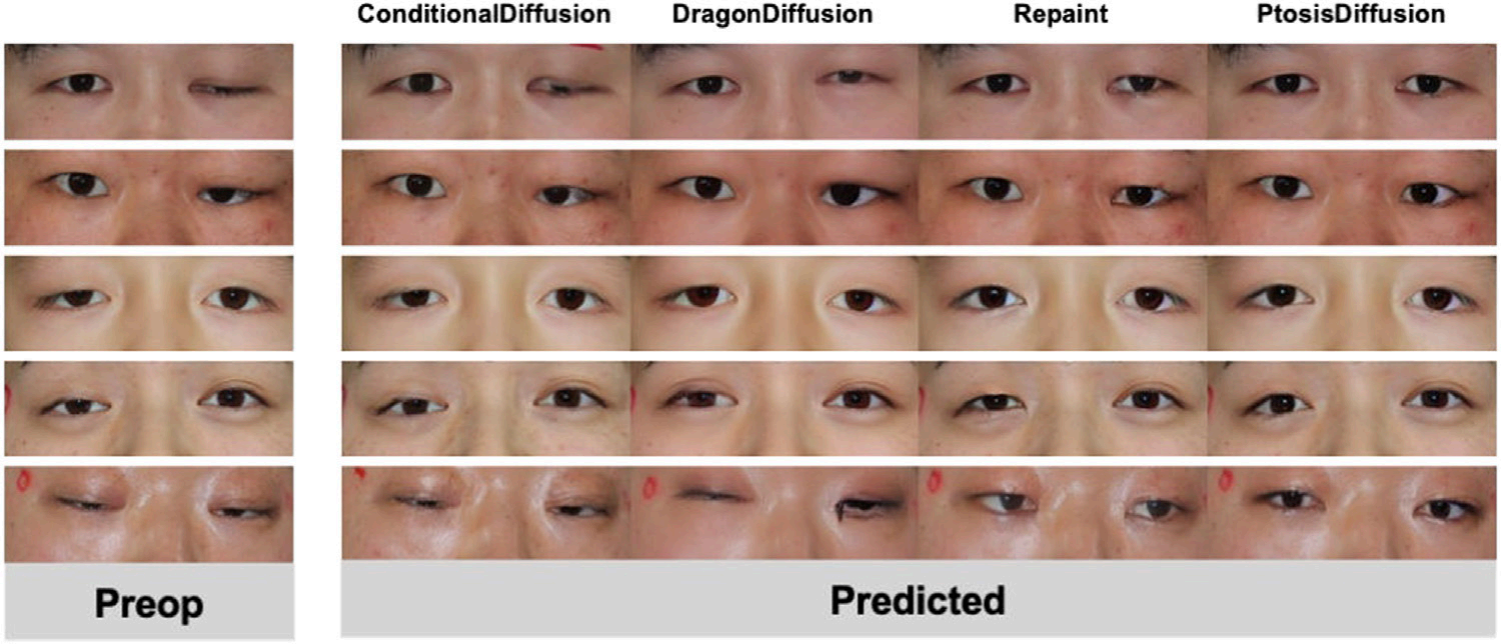
Typical examples of predicted postoperative images obtained with different models.

We collected pre-operative photos from a total of 39 patients. Among them, 13 had bilateral ptosis, 26 had unilateral ptosis, 26 were male, and 13 were female. Figure 4 displays a selection of postoperative predictions generated by different methods. A comprehensive set of all 39 predicted images is provided in the Supplementary Information (SI) for complete transparency. We conducted both human evaluation and statistical analysis to assess the performance of each method.

### 3.1 Subjective evaluation

We engaged two doctors—a junior ophthalmologist and a senior ophthalmologist—to assess the prediction results of four models across three different aspects: overall rating, correction amount, and double eyelid formation (DEF). In selecting the metrics for better evaluating prediction quality, we focused on both functional and aesthetic outcomes. The correction amount is vital for ensuring proper eyelid functionality post-surgery, which directly impacts the patient’s visual comfort and overall satisfaction. Additionally, the creation of a well-defined and persistent double-eyelid crease is of paramount importance, especially for East Asian patients (Lee et al., 2013), where aesthetic outcomes are closely tied to patient satisfaction. Therefore, double eyelid formation (DEF) was chosen as a key metric to assess the accuracy of aesthetic predictions. By incorporating both correction amount and DEF, our evaluation framework provides a comprehensive assessment of the surgical outcomes that are most relevant to patients and clinicians alike. For the overall rating, we adopted a three-point scale, evaluating and scoring in three distinct categories: 0 (poor), 1 (average), and 2 (excellent), larger value indicates better result. For correction amount, under-correction was scored as −1, over-correction as 1, and correct correction as 0, value closer to zero indicates better result. For DEF, we used a similar rating standard to the overall rating.

**FIGURE 5:**
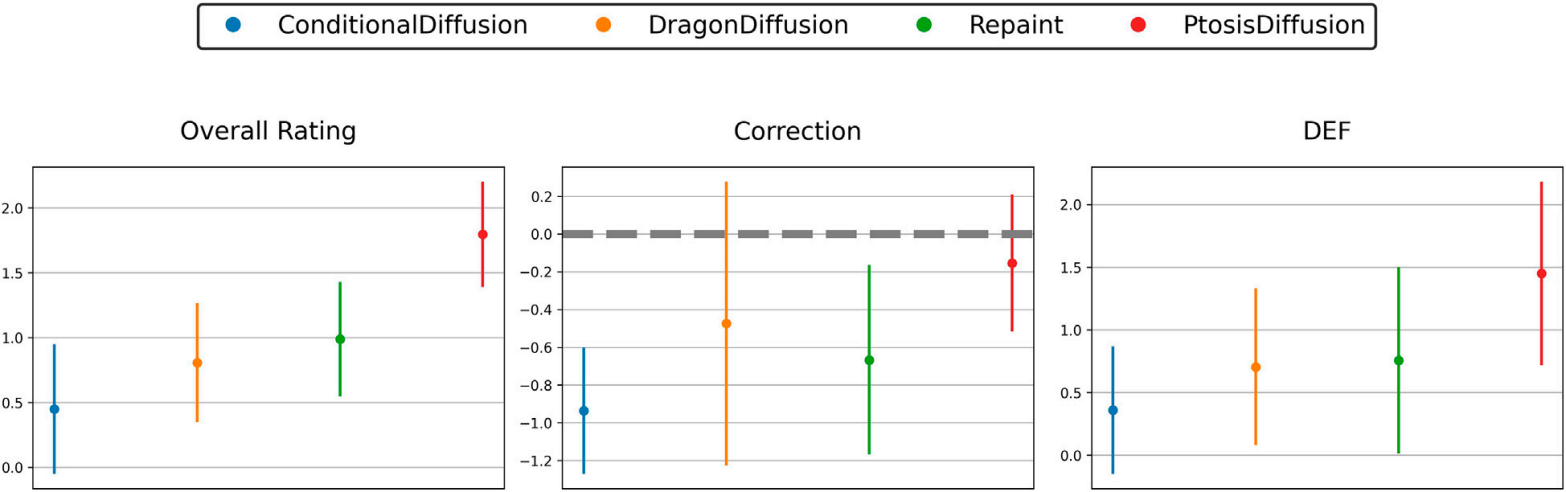
Subjective evaluation of predicted postoperative images obtained with different models: three-point scale of overall rating, correction and double eyelid formation (DEF).

The results, as shown in Table 1 and Figure 5, indicate that our PtosisDiffusion model outperformed the other three models in all aspects.

**TABLE 1:** Mean and standard deviation of evaluation metrics. For overall rating and double eyelid formation (DEF), larger value indicates better result. For correction amount, value closer to zero indicates better result.

Model	Overall ↑	Correction →0	DEF ↑
Conditional	0.45±0.50	−0.94±0.34	0.36±0.51
Dragon	0.81±0.46	−0.47±0.75	0.71±0.63
Repaint	0.99±0.44	−0.67±0.50	0.76±0.74
PtosisDiffusion	1.79±0.41	−0.15±0.36	1.45±0.73

### 3.2 Statistical evaluation

**FIGURE 6:**
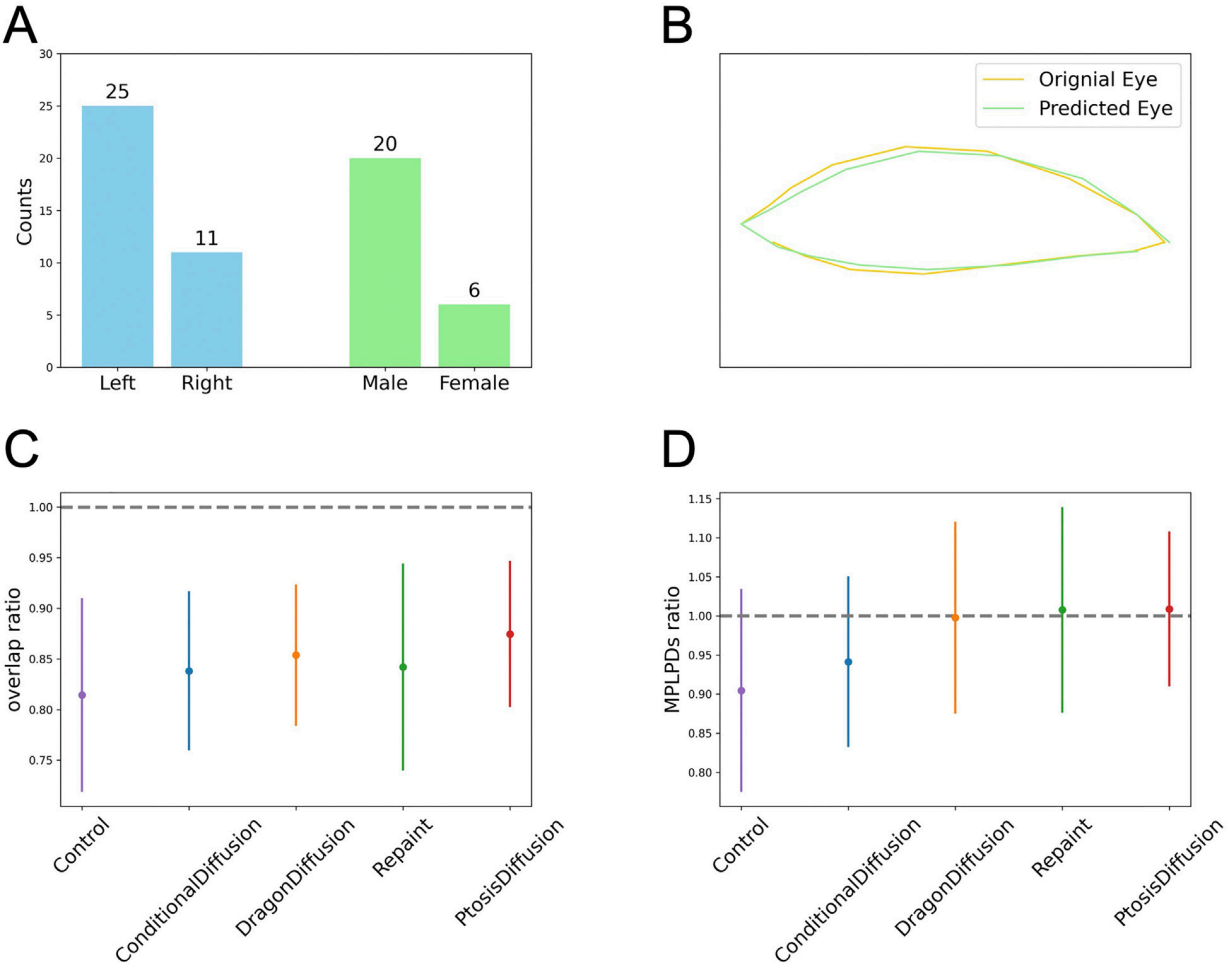
Objective assessment of the predicted postoperative images obtained with different models. **(A)** The clinical characteristics for patients with unilateral ptosis. **(B)** Illustration for eye contour and overlap ratio: the ratio of the intersection area over the union area of the predicted eye region (green) and the original eye region (yellow). **(C, D)** The plots for the overlap ratio and MPLPD ratio across the four models and the control group.

Since we only collected pre-operative images of patients, we do not have post-operative images for direct comparison. Instead, due to the symmetry of the eyes, we selected patients with unilateral ptosis and used the unaffected eye as a standard for comparison. There are a total of 26 patients, with 15 having left eye ptosis and 11 having right eye ptosis. Among them, 20 are male and 6 are female. For comparison, we first aligned the two eyes and then horizontally flipped one eye so that it overlaps with the other. We then detected the contours for both eyes, as shown in Figure 6. We use two stats for evaluation. One is overlap ratio, which is defined as the intersection area of the two eyes over the union area of the two eyes. Another metric is the MPLPD ratio, defined as the ratio of the MPLPDs of the two eyes at a given angle, θ . We select θ at increments of 15° from 0° to 360° . We also introduce a control group, which involves calculating statistics for the original, unmodified images.

As shown in Table 2 and Figures 6C, D, all four models perform better than the control group. Our model outperforms the other three models in terms of overlap ratio and performs comparably in MPLPD ratio to Dragon Diffusion and Repaint.

**TABLE 2:** Mean and standard deviation of evaluation metrics. For overlap ratio, larger values indicate better result. For MPLPD ratio, values closer to one are better.

Model	Overlap ratio ↑	MPLPD ratio →1
Control	0.81±0.10	0.90±0.12
Conditional	0.83±0.08	0.94±0.11
Dragon	0.85±0.07	1.00±0.12
Repaint	0.84±0.10	1.01±0.13
PtosisDiffusion	0.87±0.07	1.01±0.10

### 3.3 Robustness evaluation

**FIGURE 7:**
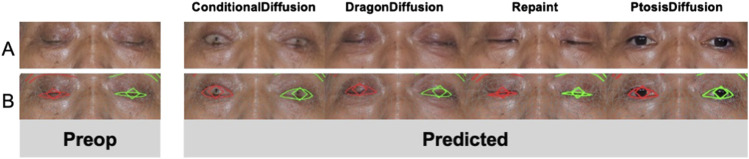
An example of predicted postoperative images obtained with different models. **(A)** Model outputs under extreme case. **(B)** Face mesh detections for test models.

We investigate post-operative predictions in extreme cases with severe ptosis, as shown in Figure 7. The other three models fail to output meaningful post-operative images. Yet face meshes are still detected for these cases, and thus output reasonable measurements based on face mesh. This elucidates why the subjective evaluation of our model demonstrates significantly superior performance compared to other models, whereas the statistical evaluation shows relatively less pronounced improvements.

### 3.4 Ablation study

**FIGURE 8:**
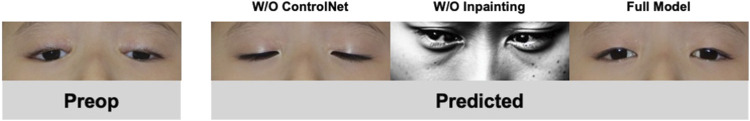
An example of input preoperative images and predicted postoperative images obtained with three different methods (w/o ControlNet, the model w/o inpainting, the full model).

In our ablation study, we conducted a series of comparisons to evaluate the individual contributions of different components within our workflow. First, we examined the results after removing ControlNet from the complete workflow. Second, we assessed the impact of excluding the inpainting component. Finally, we compared these modified workflows against the results produced by the full, unaltered workflow. The results in Figure 8 clearly demonstrate that both components are essential to our workflow.

## 4 Discussion

Our study employs a diffusion model to predict the postoperative appearance of ptosis patients using preoperative photographs. We use both statistics and subjective evaluations to assess the predicted images. To the best of our knowledge, this is the first time a diffusion model has been used for such predictions in ptosis patients.

Subjective evaluation results show that PtosisDiffusion model significantly outperformed the other three models in overall rating, correction, and double eyelid formation. This highlights the superior quality and accuracy of the images generated by our diffusion model, underscoring its potential for clinical use in predicting postoperative outcomes. Statistical evaluation shows that PtosisDiffusion model achieved the highest overlap ratio and second best MPLPD ratio. Compared to the unmodified control, there is a significant improvement, indicating that our model produces better postoperative symmetry in patients with unilateral ptosis. These findings are consistent with the results from the subjective evaluation.

The robustness evaluation reveals that our model maintains consistent performance even in extreme cases, such as fully closed eyes, where other models fail to produce reasonable reconstructions. This robustness underscores the effectiveness of our diffusion model in handling a variety of challenging scenarios, further validating its potential for reliable clinical application.

The results of our ablation studies demonstrate that each component of our model is essential. The removal of the ControlNet component results in the model losing the ability to control the eye contour of the image. Similarly, omitting the inpainting component results in a loss of identity preservation for the original patients.

Compared to studies using GAN networks, our diffusion model does not require paired data and extra training. This is a significant advantage as it reduces the complexity and resource intensity associated with data collection and preparation. The ability to generate the prediction without the need for paired preoperative and postoperative images can streamline the process and make it more feasible to implement in clinical settings. Notably, the model takes approximately 12 s to generate predictions on a Nvidia T4 GPU, which underscores its computational efficiency and feasibility for implementation in a clinical environment. Furthermore, the images generated by the diffusion model are more realistic, enhancing the visual accuracy and reliability of the predicted postoperative appearances.

An important consideration in studies involving postoperative image prediction is the quality of the preoperative images. In previous studies, image-to-image translation methods were employed, where the quality of the input preoperative image directly influenced the quality of the generated postoperative prediction. However, in our study, we utilized the inpainting approach, which is a generative process rather than a translation. This method synthesizes the postoperative appearance based on the input image’s features rather than simply altering the original image. As a result, the quality of the preoperative photograph does not significantly impact the quality of the predicted image, provided that the preoperative image is not extremely blurry—a condition that is typically avoided in clinical practice. The inpainting method we used in this study mitigates the dependency on preoperative image quality, thus ensuring the reliability and applicability of the findings. While our inpainting method offers improved robustness, certain factors may still influence the accuracy and consistency of the model’s predictions. The accuracy of the facial mesh used in the workflow is critical; if key facial landmarks are incorrectly identified, it can lead to distorted outputs. Additionally, variability in facial expressions in the preoperative images can impact the predictions, as different expressions may alter the appearance of features and affect synthesis outcomes. Furthermore, the parameters and settings applied during the inpainting process can also affect the quality of predictions. Careful optimization of these settings is essential to accommodate different input images and ensure high-quality postoperative predictions.

One limitation of our study is the lack of actual postoperative photographs for comparison, as the diffusion model does not require paired data. This absence of real postoperative images as a benchmark may impact the thorough evaluation of the model’s predictive accuracy. However, the use of unpaired data is also an advantage, as it allows for greater flexibility and applicability of the model, particularly in scenarios where obtaining paired data is challenging. Despite this limitation, we have employed statistical methods based on symmetry and overlap rates to evaluate the model’s performance. These methods, while valuable, may not fully capture the nuances of individual patient outcomes. Therefore, future work should consider integrating methods to validate the predicted images against actual postoperative results to further substantiate the model’s effectiveness. Expanding the dataset to include postoperative images will enable more comprehensive and direct comparisons, thereby enhancing the overall evaluation of the model’s predictive capabilities.

Another limitation is the lack of precise control over the generated images. While we generate canny edge maps based on the MRR value for ControlNet reference, the output results often exhibit different MRR values. We attribute this discrepancy to the inherent characteristics of the ControlNet and the diffusion model. The accuracy of ControlNet is contingent upon the precision of the canny edge detection, which may not be ideal for our specific case. Additionally, as a probabilistic model, the diffusion model may produce slightly varying results with each iteration. This variability in generated images introduces a degree of uncertainty that could be a limitation when applying the model to clinical decision-making. Future research should aim to develop methods that enhance the control over image generation, possibly by refining edge detection techniques or incorporating additional constraints to reduce output variability. If further techniques become available for precise control of the diffusion model, our model could potentially achieve even greater performance enhancements.

Despite the limitations, our study opens several promising avenues for future research and clinical applications. PtosisDiffusion could be particularly useful in preoperative planning, allowing surgeons to simulate and visualize potential outcomes based on individual patient characteristics. This capability could enhance patient counseling, helping patients set realistic expectations and making informed decisions about their surgery. Additionally, PtosisDiffusion could serve as a valuable tool in surgical training, enabling trainees to explore different surgical outcomes without actual patient involvement. Future studies could also explore the application of diffusion models in predicting outcomes for other types of ocular and facial surgeries, broadening the scope of its clinical utility. Additionally, integrating longitudinal data that captures different stages of postoperative recovery could offer more comprehensive predictions, aiding in both surgical planning and patient counseling. Moreover, the integration of longitudinal data capturing different stages of postoperative recovery could provide more dynamic and comprehensive predictions, aiding surgeons in tailoring postoperative care and optimizing recovery outcomes.

## 5 Conclusion

This study introduced a diffusion model approach to predict postoperative appearance for ptosis patients using preoperative photographs, without the need for paired data and extra training. We tested four different diffusion models, with PtosisDiffusion, producing the best results. Subjective and statistical evaluations demonstrated that PtosisDiffusion outperformed others in overall rating, correction, and double eyelid formation, achieving better postoperative symmetry.

Despite limitations such as lack of real postoperative photographs for comparison, our model showed robustness in generating satisfactory images under extreme conditions. Overall, our diffusion model, particularly PtosisDiffusion, represents a significant advancement in predicting postoperative outcomes for ptosis patients, with the potential to enhance clinical practice.

## Data Availability

The raw data supporting the conclusions of this article will be made available by the authors, without undue reservation.
