# Supplementary material for: PtosisDiffusion: a training-free workflow for precisely predicting post-operative appearance in blepharoptosis patients based on diffusion models
Source: Front Cell Dev Biol. 2024 Oct 30;12:1459336. doi: 10.3389/fcell.2024.1459336 (PMC11557522; doi:10.3389/fcell.2024.1459336)
Supplement: Supplementary file 1 [file DataSheet1.docx]

**Title: PtosisDiffusion: A Workflow for Predicting Postoperative Appearance in Blepharoptosis Patients Using Diffusion Models**

**Supplementary Information**

**Supplementary Figure. Complete Set of Postoperative Prediction Images Generated by PtosisDiffusion and Comparative Models**

**
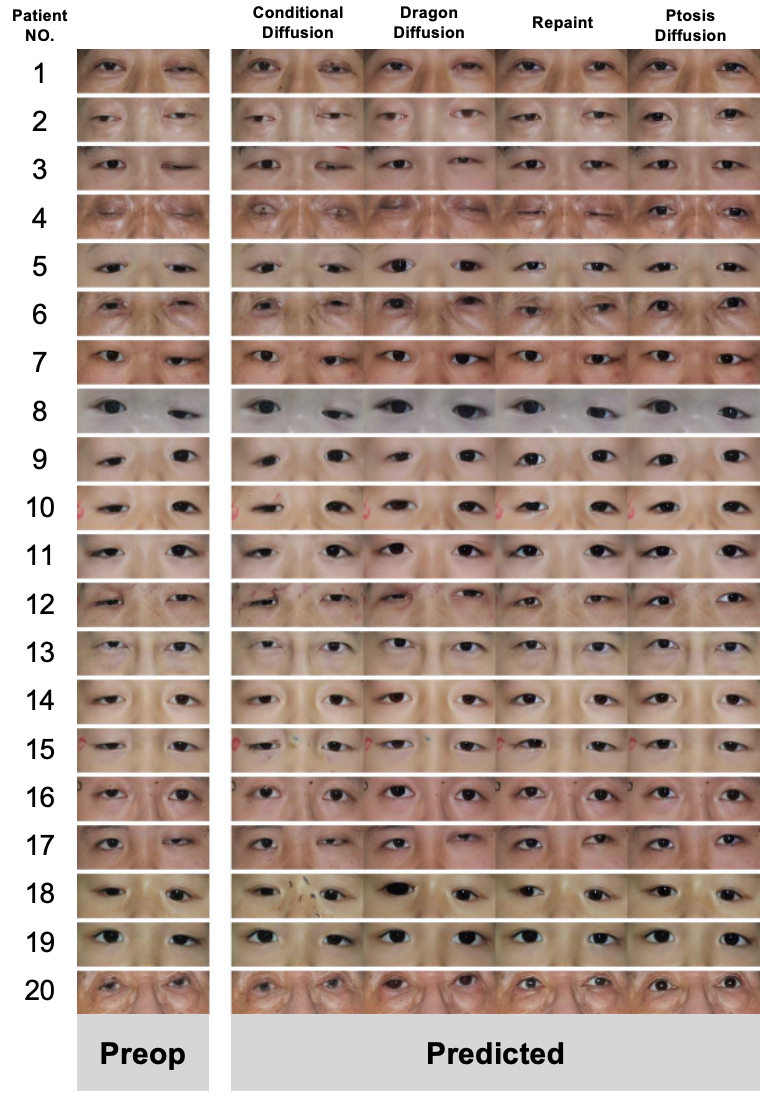
**

**
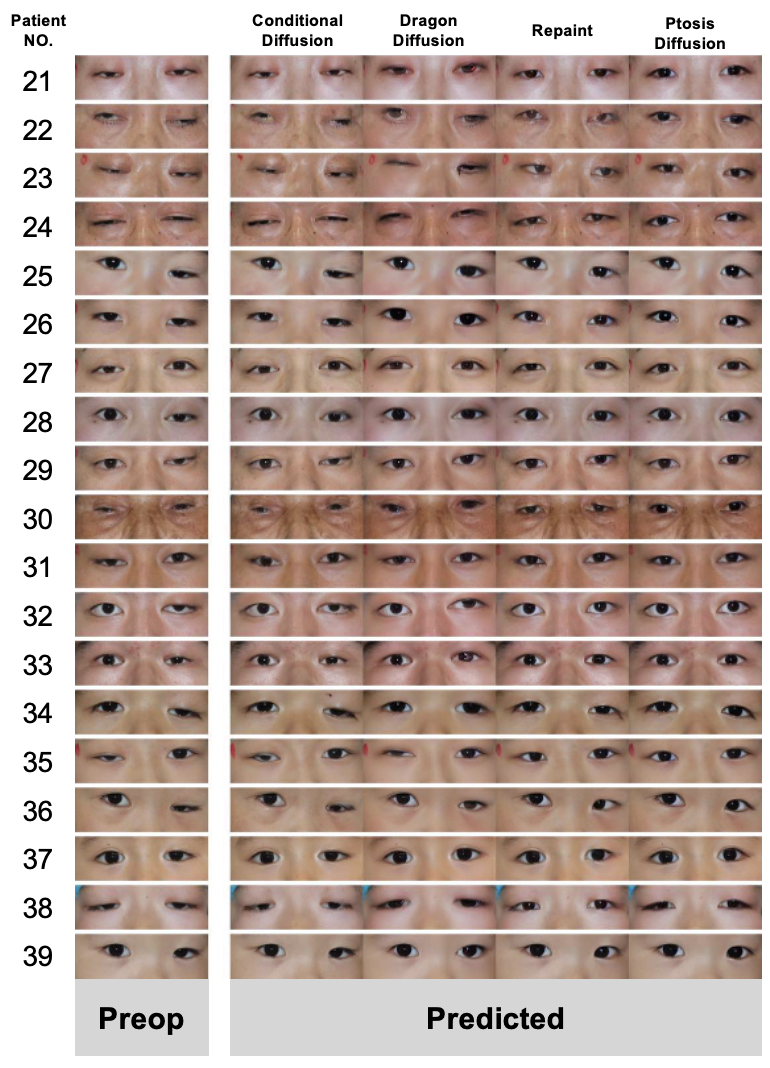
**
